# Response: Impact of implantable cardioverter defibrillator on survival in patients with nonischemic dilated cardiomyopathy

**DOI:** 10.1002/clc.24138

**Published:** 2023-08-22

**Authors:** Hossein Salehi Omran, Rana Irilouzadian, Mohammad Taghi Hedayati Goudarzi, Mohammad Taghi Salehi Omran

**Affiliations:** ^1^ School of Medicine Shahid Beheshti University of Medical Sciences Tehran Iran; ^2^ Burn Research Center Iran University of Medical Sciences Tehran Iran; ^3^ Department of Cardiology, Rohani Hospital, School of Medicine Babol University of Medical Sciences Babol Iran; ^4^ Department of Cardiology Babol University of Medical Sciences Babol Iran

## Abstract

Our previous study aimed to investigate overall survival (OS) and sudden cardiac death (SCD) variables in nonischemic dilated cardiomyopathy (DCM) patients treated only with standard medical treatments versus those who received implantable cardioverter defibrillator (ICD) in addition to routine medical treatments. Our findings revealed no significant difference in OS between the two groups (*p* = .25), but a significant decrease in SCD rate due to ICD insertion (*p* = .02). Furthermore, we found no significant difference between the two groups concerning baseline characteristics and type of medical treatments received. We attempted to answer and clarify the concerning points regarding the survival benefits of ICD insertion in nonischemic DCM patients that were mentioned.

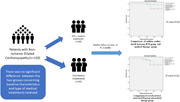

To the Editor,

We would like to thank Dr Kataoka and Dr Imamura for their interest in our study and for taking the time to express their concerns.^1^ Some concerning points were mentioned regarding survival outcomes of implantable cardioverter defibrillator (ICD) insertion in patients with nonischemic dilated cardiomyopathy (DCM) and some baseline characteristic of the patients who were investigated in our study.

In our study entitled “The impact of implantable cardioverter defibrillator on the prognosis of nonischemic dilated cardiomyopathy patients compared with standard medical treatments,” we aimed to evaluate the clinical outcomes, including overall survival (OS) and sudden cardiac death (SCD), of participants with nonischemic DCM who were only treated with standard medical treatments such as angiotensin‐converting enzyme (ACE) inhibitors and beta‐blockers compared to those who underwent ICD, in addition to the above‐mentioned routine medical treatments.^2^ As previously mentioned in the methodology section of our article, SCD was considered as mortality due to cardiac arrhythmias. OS was defined as mortalities due to all causes, such as cerebral emboli, severe heart failure, and pulmonary edema. In our study, no significant difference was observed in all‐cause mortalities between the two groups (*p* = .25); however, a significant decrease in the rate of SCD was recorded due to ICD insertion (*p* = .02).

In a randomized clinical trial (RCT) by Bänsch et al.,^3^ a total of 104 DCM patients were investigated regarding the survival beneficial outcomes of ICD insertion. Similarly, their findings showed that there was no significant difference in all‐cause mortality rates in a 1‐year or long‐term follow‐up period. Furthermore, in another RCT by Kober et al.,^4^ patients with nonischemic systolic heart failure were evaluated in terms of different survival variables. They showed that prophylactic ICD implantation was not associated with a significant decrease in mortality rate due to any cause compared to standard medical therapy (*p* = .28). However, ICD implantation significantly reduced the rate of mortality due to SCD (*p* = .005), which was consistent with our findings. Moreover, in another study by Kadish et al.,^5^ the nonischemic DCM patients were evaluated in terms of mortality variables, including SCD and all‐cause mortality, between those treated with standard medical treatments and those who underwent ICD implantation in addition to the aforementioned medical therapies. Accordingly, ICD insertion was associated with a significant decrease in SCD mortality. However, no significant difference was reported between those who underwent ICD implantation and those who received only standard medical therapies regarding deaths from any cause in that study. These findings corroborate our results.

As mentioned above, OS was regarding any potential cause of death such as pulmonary edema, severe heart failure, cardiogenic shock, pulmonary emboli, and brain stroke in which no significant influence of ICD insertion was observed. Other treatment measures like above‐mentioned medical therapies, cardiac transplantation, and healthy lifestyle might have more effect on OS rather than ICD implantation to prevent such deaths. On the other hand, ICD will significantly reduce cardiac arrhythmias leading to SCD.

We are aware that one of the main limitations of our study was the small sample size of the participants who were investigated in our study due to a lack of financial recourses in our country. We encourage more studies with a larger sample size to be performed in the near future that may yield different and more significant results.

All the patients with nonischemic DCM aged 21–85 years with left ventricular ejection fraction ≤ 35% and New York Heart Association functional class of II to IV who were referred to our hospital were included in our study. All patients with a history of acute myocarditis, congenital heart failure, or coronary artery disease were excluded. All participants investigated in this study were candidates for ICD insertion. The patients who received only medical therapies were those who refused to insert an ICD due to nonmedical reasons such as financial problems and inadequate access to medical services. On the other hand, those who cooperated well and underwent an ICD were categorized as the ICD + medical therapy group. Subsequently, a variety of baseline characteristic variables, including demographic factors, potential influential comorbidities, cardiac function variables, cardiac arrhythmias, valve disorders, and left or right bundle branch blocks, were evaluated. All of the participants were given the standard medical treatments, including ACE inhibitors or angiotensin receptor blockers, beta‐blockers (such as carvedilol and metoprolol), diuretics (such as thiazide and furosemide), aldosterone antagonists (such as spironolactone), and digoxin in those with no contraindications. There was no significant difference in the type of medical treatment between the participants who underwent ICD or not; however, a slight difference was observed between the two groups, which was inevitable due to the retrospective nature of the study.

Considering the various contraindications to prescribe the above‐mentioned drugs, beta‐blockers were prescribed with caution for those with chronic obstructive pulmonary disease, asthma, diabetes mellitus, peripheral vascular diseases, sinus bradycardia, or atrioventricular block. For those experiencing severe side effects, the prescription may have been altered and alternative treatments were considered.

Additionally, in relation to supraventricular tachyarrhythmias such as atrial fibrillation, our analysis did not reveal any significant difference between the patients who underwent ICD implantation versus those who did not (23.3% vs. 26.7%, *p* = .67). We investigated various baseline characteristics in our study that could have been mentioned in the article, but we chose to omit some of them that were not significantly different between the two groups to keep the manuscript and the included result tables as comprehensible as possible. Taken together, we tried to reduce the errors and the factors that may adversely affect our results.

## CONFLICT OF INTEREST STATEMENT

The authors declare no conflict of interest.
